# A study of Chinese law on restricting personal liberty for public health protection: taking the COVID-19 epidemic as the entry point

**DOI:** 10.3389/fpubh.2025.1670119

**Published:** 2025-10-21

**Authors:** Tengfei Liu, Zhongwu Ma

**Affiliations:** School of Law, Shandong University, Qingdao, China

**Keywords:** COVID-19, public health, personal freedom, administrative enforcement, principle of proportionality

## Abstract

During the COVID-19 pandemic, China adopted restrictive measures such as mandatory quarantine, health code management, and community lockdowns. These actions were effective in containing the epidemic but often lacked clear legal authorization or procedural safeguards, raising concerns about excessive restrictions on personal liberty. From a legal and policy perspective, this paper examines the statutory framework that enabled such measures, focusing on the Law on the Prevention and Control of Infectious Diseases and the Emergency Response Law. It argues that vague authorization and weak procedural constraints left space for local governments to exercise discretionary overreach, exemplified by excessive lockdowns and misuse of digital tools. Building on constitutional principles, the study highlights how legality, necessity, and proportionality should be fundamental limits on emergency powers. It shows that the absence of detailed procedures and rights-protection mechanisms undermined these principles, leading to conflicts between public health and human rights. The paper contributes to Chinese public health law scholarship by clarifying these institutional weaknesses and by proposing reforms to strengthen procedural guarantees, judicial oversight, and regulation of digital surveillance tools. In doing so, it advances understanding of how to balance civil liberties with collective security in future public health crises.

## 1 Introduction

The COVID-19 pandemic, as one of the most globally consequential public health crises of the 21st century, not only challenged state governance capacity but also posed severe tests to the protection of fundamental rights. In China, to prevent patients, carriers, suspected cases, and close contacts from further spreading the virus, the government implemented strict measures such as home quarantine, regional lockdowns, and digital health code management (1). Expanding public authority under emergency conditions to quickly restore social order and safeguard long-term freedoms can be considered a legitimate response (2). However, such an expansion of power must not be without boundaries. Several measures—such as welding doors shut, late-night transfers, or arbitrary assignment of health code statuses—lacked clear legal authorization and raised widespread concern regarding proportionality and legality (3).

Personal liberty, recognized by constitutions worldwide as a core human right, prohibits any illegal deprivation or restriction of freedom. In China, the principle of “locking power in the cage of institutions” has long been emphasized to constrain governmental authority and prevent encroachment on private rights (4). Yet during the pandemic, local governments frequently implemented restrictive measures without a statutory basis, highlighting the risks of administrative overreach in times of crisis.

Existing scholarship has examined the effectiveness of epidemic control and the general conflict between public health and civil liberties. However, there remains a research gap in systematically analyzing how China’s legal framework—particularly the vague authorization in public health legislation and the reliance on “soft law” instruments such as health codes—affects constitutional safeguards during emergencies. This review seeks to fill that gap by critically evaluating the legal foundations of restrictive measures, assessing the principles of legality, necessity, and proportionality in practice, and proposing concrete reforms. In doing so, it advances debates on how to balance state intervention powers with the protection of fundamental rights within China’s public health law framework (5).

## 2 The legal basis and temporary measures for restricting personal freedom

In responding to major public health emergencies, the exercise of state power is indispensable. Yet for a country governed by the rule of law, the key issue is how to reconcile scientific prevention with legal limits on governmental authority. During the COVID-19 pandemic, China relied on both statutory authorizations—such as mandatory isolation—and temporary policy measures, including health codes, travel history cards, and community lockdowns. While these measures contributed to curbing transmission, their scope and intrusiveness also generated widespread controversy, requiring closer legal scrutiny.

### 2.1 The legal basis for restricting personal freedom in public health emergencies

China’s current legal system provides a foundation for restricting personal freedom in emergencies through instruments such as the Administrative Compulsion Law, the Law on the Prevention and Control of Infectious Diseases, the Emergency Response Law, the Public Security Administration Punishments Law, and the Frontier Health and Quarantine Law. These provisions authorize compulsory measures such as isolation, lockdowns, and temporary detention (6). In principle, such laws establish the legitimacy of administrative responses and ensure a legal basis for crisis management.

However, most of these authorizations remain highly general and principle-based, lacking detailed boundaries, operating procedures, and relief mechanisms. For example, while the Infectious Diseases Law allows lockdowns, it does not specify procedural requirements for decision-making or avenues for citizens to contest such restrictions. This legal vagueness enabled broad discretion, leading local authorities to bypass statutory safeguards under the pretext of “emergency.” The 2022 Zhaoyang District case illustrates how neighborhood committees unilaterally expanded lockdown measures without explicit authorization, exposing the vacuum between legal norms and policy practice (7).

By contrast, the European Union upholds the principle of “explicit statutory authorization” in regulating emergency measures, requiring that the scope, conditions, actors, and procedures for restricting freedom be precisely defined in written law, with judicial review strictly confining administrative power within the limits of authorization. The United States, in turn, centers on the doctrine of the “least restrictive means,” establishing a tiered review system: when measures involve core constitutional rights such as freedom of speech or religion, strict scrutiny applies, obliging the government to prove that the measure is both the only and the least restrictive option, failing which it will be deemed unconstitutional; for general public administration measures, the standard of review is relatively relaxed but still examines necessity. In comparison, although China has enacted multiple laws granting emergency response powers, these provisions remain largely at the level of general principles, lacking detailed procedural design and clear boundaries. This grants administrative organs excessive discretion, as illustrated by local authorities expanding lockdown measures during the pandemic without clear authorization—an example of power abuse stemming from legal ambiguity. Overall, China still needs to further refine its legal framework to restrict administrative discretion and strengthen rights remedies, to achieve a balance between public interests and individual rights.

### 2.2 Temporary measures to restrict personal freedom in a public health emergency

In addition to statutory authorizations, local governments widely adopted informal governance tools such as health codes, itinerary cards, and community lockdowns. While efficient in practice, these measures lacked legislative grounding and relied heavily on administrative discretion, raising concerns of disguised compulsory action beyond legal oversight.

*Health codes and itinerary cards*—Initially designed as innovative tools to classify risk levels and track movement (8). These systems gradually revealed problems such as opaque algorithms, arbitrary “red code” assignments, and a lack of error-correction channels (9). In some instances, they were misused as tools of social control, sparking controversy over legality and proportionality.

*Community lockdowns*–Frequently implemented through community notices, these restrictions effectively contained transmission but often neglected the basic needs of residents. Incidents such as the death of a four-month-old infant in Henan during centralized quarantine illustrate the dangers of rigid, “one-size-fits-all” enforcement that prioritizes control over rights protection (10).

Although these temporary measures have, to some extent, filled governance gaps, their weak legal basis and lack of independent review mechanisms have left citizens vulnerable to arbitrary restrictions. By contrast, the European Union has established strict normative conditions for contact tracing applications, requiring compliance with the principles of necessity and temporal limitation, while ensuring codified safeguards through the existing data protection framework, such as the General Data Protection Regulation (GDPR). This institutionalized design not only delineates clear legal boundaries but also provides stronger avenues of redress for the protection of citizens’ rights.

### 2.3 Assessment

China’s legal system provided a formal foundation for emergency measures but failed to define clear procedural limits or oversight mechanisms. This gap between law and practice enabled local overreach and fostered reliance on “soft law” tools without statutory legitimacy. Comparative experience demonstrates that without explicit legal standards and judicial checks, temporary measures risk undermining constitutional safeguards. Section 4 will further examine how these deficiencies translated into concrete abuses during implementation.

## 3 The norms and principles safeguarding personal freedom rights

Personal freedom has long been recognized as one of the most fundamental civil rights in governance systems (11). During public health emergencies, epidemic control often necessitates temporary restrictions on freedom, placing higher demands on the rule-of-law framework and rights protection system. Clarifying the legal norms and applicable principles is crucial not only to evaluate the legality and rationality of such measures but also to balance public interests and individual rights.

### 3.1 Normative content of the protection of personal freedom rights

China’s Constitution explicitly stipulates in Article 37 that no citizen shall be unlawfully deprived of personal freedom. The Administrative Compulsion Law further requires that restrictions on freedom must be authorized by legislation. Thus, epidemic control must not bypass statutory boundaries. This establishes a baseline: even in crises, the exercise of state power must remain within the legal framework.

In theory, Gostin identifies two legal principles underpinning isolation powers: the principle of “no harm to others” and the principle of “public safety first.” (12) These provide governments with a legitimate basis to implement compulsory isolation, restrict gatherings, or temporarily close venues. Yet in the Chinese context, the pressing challenge is not the absence of statutory norms, but rather the tendency of local authorities to expand administrative power under the justification of necessity. While the law allows emergency intervention, over-extension beyond statutory authorization undermines legality and erodes citizens’ trust.

For example, in the September 2021 Hebei case where officials tied an older adult to a tree for violating lockdown orders, the measure grossly exceeded lawful authority. Such incidents demonstrate that epidemic governance can slip into arbitrary enforcement, violating human dignity and contravening constitutional protections. They also expose a deeper structural problem: the gap between legal norms and grassroots enforcement practices. Although China’s central laws emphasize legality, proportionality, and respect for human rights, enforcement often reflects local discretion and performance-driven governance.

Therefore, while public interests may justify certain concessions of personal freedom, these concessions must remain circumscribed by law. Measures that exceed statutory authority not only constitute abuses of power but also risk damaging the legitimacy of epidemic governance and the broader image of the rule of law (13).

### 3.2 The principal content of safeguarding the freedom rights of people

Legal norms must be implemented in light of guiding principles, which define both the bottom line and the direction of rights protection. Three principles stand out: the rule of law, proportionality, and due process.

The rule of law principle requires all restrictions to be imposed strictly in accordance with legislation, ensuring fairness and justice. Proportionality demands that state interventions be appropriate, necessary, and minimally restrictive (14). The due process principle requires transparent and impartial procedures to guarantee that rights are not curtailed arbitrarily (15).

In practice, the proportionality principle is often the most vulnerable. In China, lockdowns, travel bans, and quarantine measures have often been enforced rigidly and uniformly, prioritizing administrative efficiency over individual circumstances. Instead of adopting the least restrictive means, local authorities often default to blanket restrictions. While such measures may appear effective, they risk exceeding what is necessary to achieve public health objectives, thereby violating the principle of proportionality (16).

Due process faces similar difficulties. Legally, only administrative or judicial organs may authorize restrictions on personal freedom. However, during the COVID-19 pandemic, hospitals and medical staff at times unilaterally imposed isolation (17). These actors, lacking expertise in administrative law, were unable to assess legality or proportionality. This reflects a systemic problem of “authority misplacement,” where entities without statutory power make de facto decisions restricting personal freedom. Such practices undermine the constitutional requirement that coercive measures be carried out only by legally competent organs.

Furthermore, although rights may be restricted for public health, they cannot be suspended. The minimum requirements of rights protection must always be respected (18). Yet selective enforcement, discretionary management, and *ad hoc* local regulations remain common in grassroots governance. This demonstrates a disconnect between central legal mandates and local enforcement realities.

### 3.3 China’s enforcement challenges

China’s legal framework does contain the necessary principles and statutory norms to regulate restrictions on freedom in emergencies. The real challenge lies in law enforcement and implementation. Unlike jurisdictions with strong traditions of judicial review, China relies heavily on administrative bodies to execute emergency governance. This institutional design risks over-concentration of power, with limited channels for independent oversight.

Three enforcement challenges are particularly prominent: Local government overreach. Driven by epidemic control targets, local authorities often adopt “one-size-fits-all” measures that prioritize efficiency over legality. This creates a structural risk of excessive restrictions and abuse of power. Procedural legitimacy deficits. Many restrictions lack independent review or accessible remedies. Citizens often have no effective means to challenge disproportionate measures, weakening trust in the system. Central-local enforcement gap. While central laws emphasize legality, proportionality, and due process, these principles are diluted in grassroots practice. The tension between centralized legal norms and localized performance pressures results in inconsistent protection of citizens’ rights.

Addressing these challenges requires not only codified laws but also institutional safeguards: strengthening procedural review, clarifying authority boundaries, and introducing accountability mechanisms. Only by embedding rights protection into the enforcement process can epidemic governance in China achieve both efficiency and legitimacy.

Safeguarding personal freedom during epidemics requires more than rhetorical adherence to principles. In China, the challenge lies in transforming constitutional and statutory guarantees into operational norms that can guide grassroots actors. Ensuring that only legally authorized entities may impose restrictions, embedding proportionality review, and strengthening due process mechanisms are essential to avoid arbitrary or abusive practices. Upholding the bottom line of rights protection is not only a legal requirement but also the foundation for sustaining social trust and the credibility of the rule of law during public health crises.

## 4 Application issues regarding the restriction of personal freedom in public health emergencies

During public health emergencies, restrictive measures adopted by the government may carry a certain degree of legitimacy and necessity in safeguarding collective safety. However, China’s COVID-19 practices revealed recurring abuse of power, procedural irregularities, and overreliance on technological tools. These led to excessive restrictions on personal liberty and created profound concerns for both society and the rule of law. To highlight the structural weaknesses, this section classifies the problems into three dimensions—procedural abuses, technological distortions, and discretionary overreach—and then assesses the gap between de jure and de facto practices.

### 4.1 Procedural abuses in isolation and lockdown measures

Isolation and lockdown measures such as “city closures” and “community blockades” proved effective in curbing transmission, yet at the grassroots level, their implementation often deviated from legal requirements. Problems included unclear authorization, missing procedures, and unauthorized entities acting as decision-makers.

Excessive blockades: Neighborhood committees and village committees sometimes closed unit doors or even blocked fire exits, seriously endangering residents’ safety (19). These practices illustrate how administrative convenience was prioritized over procedural safeguards, undermining both legality and public trust.

Factual detentions: In Hunan, a deliveryman was quarantined for 14 days after entering a community for only 2 min. Such incidents expose the absence of necessity and proportionality review, transforming epidemic prevention into arbitrary deprivation of liberty.

Emergency failures: The Urumqi fire tragedy, in which ten lives were lost, highlighted how overzealous lockdowns obstructed emergency rescue and turned public health policy into “people-prevention policy” (20). The public widely questioned whether the pursuit of zero-COVID had overridden the government’s basic duty to protect life.

From a legal standpoint, such mass lockdowns cannot be strictly categorized under Article 39 of the Infectious Diseases Law, which targets confirmed or suspected cases. Nor do they satisfy procedural safeguards under the Emergency Response Law, which requires official announcements, transparency, and review mechanisms (21). In practice, however, restrictions were often issued via informal notices from grassroots organizations. This reliance on quasi-administrative acts reflects a structural vacuum between statutory procedures and actual enforcement.

### 4.2 Technological distortions of health code applications

Health codes were initially designed as innovative tools for efficient epidemic monitoring, integrating nucleic acid results and travel data. In the early stages, they facilitated risk classification and enabled the reopening of work and travel. However, their functions gradually drifted from health management toward instruments of administrative control.

*Political misuse*: The 2022 Zhengzhou “red code” incident revealed how officials deliberately manipulated codes to prevent depositors from petitioning. This constituted not only a violation of freedom of movement but also a breach of citizens’ information rights (22). The case illustrated how digital infrastructures, once politicized, can easily be repurposed for social control.

*Discrimination against the older adults*: In Dalian, an older citizen without a smartphone was barred from entering the subway. This reflects how the uniform imposition of digital measures disproportionately harmed vulnerable groups, raising questions of equality and non-discrimination (23).

*Lack of accountability*: Arbitrary “red code” assignments without explanation or appeal channels revealed the absence of procedural due process. Citizens were left without remedies, fostering distrust in governance.

Although the central government later introduced the “nine prohibitions” to restrict misuse, these measures remained policy-level guidance rather than statutory codification (24). Consequently, health codes remained vulnerable to discretionary manipulation, underscoring the urgent need to embed legal safeguards, algorithmic transparency, and independent oversight in the use of technological tools during emergencies.

### 4.3 Discretionary overreach and local “over-implementation”

Another recurring issue was the over-extension of local discretion under the banner of epidemic control. Delegating broad responsibilities to grassroots organizations—such as neighborhood and village committees—resulted in quasi-administrative actions without formal authority, blurring the line between self-governance and state enforcement.

Examples include malicious door sealing, blanket quarantine of entire buildings, and arbitrary decisions by local cadres. These measures not only exceeded statutory authorization but also failed to meet proportionality requirements, as they imposed severe burdens on residents without individualized risk assessment. The phenomenon reflects the tendency of local authorities to “over-implement” central directives to demonstrate compliance, sometimes at the expense of rights protection.

Such practices reveal a deeper structural problem: when statutory frameworks lack precision, local governments fill the void with discretionary improvisation. However, this “governance by overreach” undermines both the legitimacy of public health measures and the credibility of the rule of law.

### 4.4 Assessment: de jure versus de facto practices

The above incidents reveal a systemic gap between de jure authority—which restricts liberty only in clearly defined situations and by authorized entities—and de facto practices, where grassroots organizations imposed sweeping measures without a proper legal basis.

The lack of procedural safeguards, transparency, and independent oversight transformed legitimate emergency powers into arbitrary restrictions. While China’s legal framework formally recognizes the need for proportionality and legality, its absence of detailed standards and accountability allowed local overreach.

Comparative experience suggests that embedding statutory limits, judicial review, and rights remedies is crucial to avoid the “policy drift” from public health protection to authoritarian control. Without such institutional safeguards, future crises risk repeating the same pattern of emergency-driven rights erosion.

## 5 The protection mechanism for personal freedom in the post-epidemic era

The COVID-19 pandemic has exposed the serious asymmetry in the relationship between power and rights in China’s emergency governance system. Under the policy of extreme epidemic prevention and control, citizens’ freedom is easily treated with marginalization, resulting in legal gaps and blind spots in rights. Entering the post-epidemic era, rethinking the legal mechanism that can protect citizens’ freedom and regulate administrative power has certain guiding significance for other public health security incidents that may occur in the future.

### 5.1 Strengthening procedural legitimacy

While temporary restrictions on freedom are sometimes necessary during public health emergencies, they must be embedded within a framework of procedural guarantees. Without such a framework, even well-intentioned restrictions risk degenerating into arbitrary enforcement. The 2025 revision of the Law on the Prevention and Control of Infectious Diseases was an important step forward, as it introduced clearer limitations on both the scope and duration of isolation measures. By codifying maximum time limits and clarifying procedural steps, the revision attempted to close the loopholes that had previously allowed indefinite or excessive restrictions. Yet despite this progress, practice during the pandemic demonstrated that many disputes—such as the arbitrary discoloration of health codes, community lockdowns lacking statutory authorization, and compulsory centralized isolation—arose because grassroots organizations were making decisions outside of their authority. Citizens often lacked any institutional channel through which to contest such restrictions (25).

To fill this gap, a unified objection and review mechanism should be created. This mechanism would require that: Any restrictive measure must be issued by an organ with formal legal authority, not by grassroots entities such as village committees or neighborhood committees. The decision-making authority must provide an official written document setting out the legal basis, reasons, and duration of the measure, while simultaneously informing affected individuals of their rights to appeal or request review (26). Fast-track procedures for review should be institutionalized so that complaints can be processed in real time by administrative or judicial bodies. This would replace the current reliance on “media exposure” or public opinion pressure as a substitute for formal legal remedies, ensuring that rights are protected through institutionalized rather than *ad hoc* means.

Embedding these guarantees would significantly enhance transparency, accountability, and predictability in emergency governance. It would also help prevent abuses such as “arbitrary code assignment” or excessive lockdowns, ensuring that restrictive measures remain consistent with legality, necessity, and proportionality (27).

### 5.2 Enhancing supervision and accountability

Another weakness exposed by the pandemic was the lack of effective oversight over local governments, which were entrusted with broad discretionary authority under the principle of territorial responsibility. This governance model allowed for rapid responses to evolving local conditions but also encouraged overreach and inconsistency across regions. To prevent future abuses, it is essential to establish a multi-layered supervision system.

First, the boundaries between the central and local authorities in emergency response must be clarified through legislation. In practice, local governments frequently issued measures beyond their statutory authority, using instruments such as “administrative notices” or “red-headed documents.” For example, some regions arbitrarily extended lockdown cycles or imposed mandatory centralized isolation on asymptomatic individuals. Such measures often lacked clear authorization in national legislation and were issued without proper procedural safeguards. Statutes should therefore explicitly delineate the scope of local emergency powers, limiting discretion and ensuring that all emergency measures are grounded in law rather than administrative convenience (28).

Second, the system of people’s congresses should be mobilized to provide substantive oversight over emergency administrative acts. Local people’s congresses should be empowered to review the legality of measures such as lockdowns, isolation orders, and health code applications. At the national level, the establishment of a Public Health Legal Review Committee under the National People’s Congress could ensure consistency by creating a centralized filing and evaluation mechanism for emergency orders. Such a mechanism would not only improve legislative oversight but also create institutional channels for citizens to challenge unlawful restrictions.

Third, accountability mechanisms must be institutionalized to deter arbitrary exercises of power. Officials who impose unauthorized or excessive restrictions—such as sealing buildings, manipulating health codes for non-health purposes, or implementing blanket quarantines—should be held liable through administrative sanctions and, where serious harm results, through criminal prosecution. Accountability must be tied to violations of the principles of proportionality and due process that result in tangible harms (29). This creates what may be called an “accountability loop,” ensuring that decision-makers bear direct consequences for unlawful acts and thereby encouraging prudence and restraint in future emergencies.

### 5.3 Codifying limits on digital surveillance tools

Digital tools such as health codes and travel history cards played a central role in epidemic governance. By integrating nucleic acid test results, travel trajectories, and contact histories, these tools enabled rapid monitoring and classification of public health risks. At the early stages of the pandemic, their efficiency was widely recognized. However, in the absence of statutory codification, these tools were highly vulnerable to misuse. The most notorious case was the 2022 Zhengzhou “red code” incident, where depositors attempting to petition over frozen bank funds found their health codes arbitrarily turned red. This political misuse of digital tools not only violated the right to freedom of movement but also undermined public confidence in the legitimacy of epidemic governance. Similarly, in Dalian, older citizens without smartphones were barred from taking the subway, illustrating how “one-size-fits-all” digital mandates disproportionately harmed vulnerable groups.

To prevent such distortions, future reforms should codify the governance of digital surveillance tools. Specifically, legislation should:

- Legally define the scope, duration, and exit mechanisms for the use of health codes, ensuring that they expire once the emergency ends.
- Require transparency in algorithm design and mandate independent third-party audits, thereby ensuring accountability and reducing risks of arbitrary or discriminatory application.
- Integrate health code governance into the framework of the Personal Information Protection Law, prohibiting any secondary use of health data for non-health purposes.

These measures would prevent the functional alienation of digital tools, ensuring that their use remains limited to epidemic prevention and does not spill over into broader mechanisms of social control. In this way, both freedom of movement and privacy rights can be protected.

## 6 Conclusion

This study has examined the legal dilemmas surrounding restrictions on personal freedom during the COVID-19 pandemic, focusing on both statutory measures authorized under existing law and policy-driven practices implemented at the grassroots level. The analysis demonstrates that while China’s legal framework—such as the Law on the Prevention and Control of Infectious Diseases and the Emergency Response Law—provides a general basis for restricting liberty in public health emergencies, the absence of detailed procedural safeguards, clear limits on duration and scope, and robust rights relief mechanisms created significant risks of administrative overreach. The misuse of technological tools, such as arbitrary health code assignments, further revealed a governance gap between efficiency-driven digital control and the constitutional protection of individual rights.

The key contribution of this study lies in clarifying the tension between public health imperatives and constitutional guarantees of liberty in China’s emergency governance system. It emphasizes that personal freedom, even in crises, cannot be treated as an inalienable right but must remain protected through law-based restrictions, transparent procedures, and effective remedies.

Looking ahead, several reform priorities emerge. In the short term, China should establish unified objection procedures and ensure that only legally authorized entities can impose restrictions on liberty. In the medium term, legislative oversight by people’s congresses and stronger accountability mechanisms for local governments should be institutionalized to prevent excessive or unauthorized measures. In the long term, a comprehensive legal framework for the governance of digital surveillance tools must be codified, embedding transparency, proportionality, and exit mechanisms to prevent functional alienation.

Ultimately, the pandemic experience underscores that safeguarding public health and protecting personal freedom are not mutually exclusive goals. Rather, the rule of law requires that state interventions be proportionate, procedurally legitimate, and open to review. By integrating procedural legitimacy, supervision, and accountability, and technological regulation into the legal architecture of emergency governance, China can construct a system that not only ensures collective security but also secures the fundamental rights and dignity of its citizens in the face of future crises.
